# Cholecystokinin: An Excitatory Modulator of Mitral/Tufted Cells in the Mouse Olfactory Bulb

**DOI:** 10.1371/journal.pone.0064170

**Published:** 2013-05-15

**Authors:** Jie Ma, Luba Dankulich-Nagrudny, Graeme Lowe

**Affiliations:** Monell Chemical Senses Center, Philadelphia, Pennsylvania, United States of America; Baylor College of Medicine, United States of America

## Abstract

Cholecystokinin (CCK) is widely distributed in the brain as a sulfated octapeptide (CCK-8S). In the olfactory bulb, CCK-8S is concentrated in two laminae: an infraglomerular band in the external plexiform layer, and an inframitral band in the internal plexiform layer (IPL), corresponding to somata and terminals of superficial tufted cells with intrabulbar projections linking duplicate glomerular maps of olfactory receptors. The physiological role of CCK in this circuit is unknown. We made patch clamp recordings of CCK effects on mitral cell spike activity in mouse olfactory bulb slices, and applied immunohistochemistry to localize CCK_B_ receptors. In cell-attached recordings, mitral cells responded to 300 nM –1 µM CCK-8S by spike excitation, suppression, or mixed excitation-suppression. Antagonists of GABA_A_ and ionotropic glutamate receptors blocked suppression, but excitation persisted. Whole-cell recordings revealed that excitation was mediated by a slow inward current, and suppression by spike inactivation or inhibitory synaptic input. Similar responses were elicited by the CCK_B_ receptor-selective agonist CCK-4 (1 µM). Excitation was less frequent but still occurred when CCK_B_ receptors were blocked by LY225910, or disrupted in CCK_B_ knockout mice, and was also observed in CCK_A_ knockouts. CCK_B_ receptor immunoreactivity was detected on mitral and superficial tufted cells, colocalized with Tbx21, and was absent from granule cells and the IPL. Our data indicate that CCK excites mitral cells postsynaptically, via both CCK_A_ and CCK_B_ receptors. We hypothesize that extrasynaptic CCK released from tufted cell terminals in the IPL may diffuse to and directly excite mitral cell bodies, creating a positive feedback loop that can amplify output from pairs of glomeruli receiving sensory inputs encoded by the same olfactory receptor. Dynamic plasticity of intrabulbar projections suggests that this could be an experience-dependent amplification mechanism for tuning and optimizing olfactory bulb signal processing in different odor environments.

## Introduction

The peptide hormone cholecystokinin (CCK) was originally described in the gastrointestinal system, and subsequently found to be abundantly expressed in the central nervous system [1]. Cell-specific post-translational cleavage of the CCK prohormone generates several bioactive fragments of different lengths [2]. The shortest of these is the sulfated carboxy-terminal octapeptide (CCK-8S), the major form produced and released in the brain [3], [4]. It has widespread central distribution including cerebral cortex, striatum, hippocampus, amygdala, thalamus and hypothalamus [5]–[7], and it serves diverse functions as a co-transmitter or modulator of neuronal activity in local circuits [8]–[16]. In the olfactory system, CCK octapeptide was initially detected in porcine, guinea pig and rat olfactory bulbs by radioimmunoassay and immunocytochemistry [3], [7], [17]. More detailed immunochemical and in situ hybridization studies showed differential localization to specific cell populations or cell layers in the rat olfactory bulb [18]–[22]. In particular, strong CCK-like immunoreactivity occurs in a subpopulation of superficial or middle tufted cells, which are bulb output neurons concentrated mostly in the distal, infraglomerular part of the external plexiform layer (EPL). A second band of heavy CCK immunoreactivity is comprised of peptidergic fibers and terminals in the inner plexiform layer (IPL), beneath a deeper layer of output neurons, the mitral cells. Sparse CCK labeling is also present in some periglomerular and deep short axon cells, and there is diffuse labeling of fibers in the granule cell layer. A similar laminar distribution of CCK immunoreactivity has been found in mouse olfactory bulb [23]–[25]. A conserved pattern of expression in superficial tufted cells and the IPL suggests a special role for CCK in the bulb circuitry. Tracer studies have revealed that CCK immunoreactive axons in the IPL originate from superficial tufted cells and comprise an intrabulbar association system linking medial and lateral halves of the bulb [26], [27]. This long range wiring is thought to form precise links between cells associated with isofunctional, mirror image glomeruli receiving sensory input encoded by the same olfactory receptor [28], [29].

Although the neuroanatomy of CCK peptide in the olfactory bulb has been well characterized, its physiological functions are unknown. The presence of CCK in superficial tufted cells and their intrabulbar projections suggests that the peptide is released at synapses that coordinate neuronal activity of linked pairs of glomeruli [27], [28]. Electron microscopy of the IPL showed that biocytin-labeled fibers of superficial tufted cells contacted dendritic processes with GABA-positive immunogold staining probably belonging to granule cells. It was hypothesized that CCK might be released from these synapses as a cotransmitter alongside glutamate to promote granule cell depolarization, leading to increased GABAergic inhibition of mitral cells [27]. However, the identity and localization of the CCK receptors mediating such actions is not known. Evidence for CCK receptors in the olfactory bulb has come mostly from CCK peptide autoradiography [30]–[36], which detected varying degrees of binding in all layers of the bulb in a variety of species, including humans [37]. Two subtypes of G-protein coupled receptors, CCK_A_ and CCK_B_ ( = CCK_1_ and CCK_2_), can bind CCK peptides and mediate their effects [38]. In the brain, the major receptor type expressed is CCK_B_, and to a lesser extent CCK_A_ [39]. In rat olfactory bulb, immunoreactivity to both CCK_A_ [40], [41] and CCK_B_ receptors [42] was found. CCK_A_ receptor-like immunoreactivity was reported in the lateral olfactory tract [41], which contains axons of mitral and tufted cells, and *in situ* hybridization detected a weak signal from CCK_A_ mRNA in the mitral cell layer and EPL [43]. Localization of the two receptor subtypes among different classes of olfactory bulb neurons has not been documented.

Here we show that exogenously delivered CCK can modulate mitral cell activity by exciting or suppressing action potential firing. We applied pharmacological and genetic approaches to dissect contributions of synaptic transmission and different CCK receptor subtypes to this modulation, and present immunohistochemical evidence that CCK_B_ receptors are expressed on mitral cell bodies. Our findings suggest an alternative pathway for CCK signaling between tufted cells and mitral cells.

## Materials and Methods

### Electrophysiology

All experiments utilized adult male and female mice from young mice (P14–21) belonging to the following strains: CD-1 (Charles River Laboratories); 129-Cckar^tm1Kpn/J^ (homozygous, CCK_A_ receptor targeted mutation) and 129-Cckbr^tm1Kpn/J^ (homozygous, CCK_B_ receptor targeted mutation) (Jackson Laboratory, Bar Harbor, ME). Mutant mouse colonies were established from breeder pairs, and genotyped according to recommended PCR protocols of Jackson Laboratory to select individuals homozygous in the targeted genes (Transnetyx Inc., Cordova, TN). Mice were maintained in a temperature-, humidity- and light cycle- controlled facility with ad libitum access to food and water. Horizontal olfactory bulb slices (360 µm) were prepared as previously described [44], [45], and viewed on an Olympus BX50WI upright microscope under differential interference contrast with a LUMPlan Fl/IR 60× NA 0.9 water immersion objective. Slices were perfused (5 ml/min) at 23°C with a bath of oxygenated artificial cerebrospinal fluid (aCSF) composed of (mM): 124 NaCl, 2.5 KCl, 26 NaHCO_3_, 1.25 NaH_2_PO_4_, 10 D-glucose, 2 CaCl_2_, 1.3 MgCl_2_. CCK receptor agonists, antagonists, and blockers of synaptic transmission were dissolved in aCSF and introduced into the bath by switching gravity-fed lines from different solution reservoirs. The time course of solution switch was calibrated by measuring the junction current of an open pipette inserted in a slice, recorded during wash-in and wash-out of aCSF with 50 mM added KCl, and computing the change in K^+^ concentration using the Nernst equation. The delay between valve switch and initiation of junction current response was estimated at 80 s. Reagents used were: CCK-8S, CCK-4 (Bachem, Torrance, California), 5,7-dichlorokynurenic acid (dCK), DL-2-amino-5-phosphonopentanoic acid (APV), 2,3-dioxo-6-nitro-1,2,3,4-tetrahydrobenzo[f]quinoxaline-7-sulfonamide (NBQX), 6-cyano-7-nitroquinoxaline-2,3-dione (CNQX), bicuculline methiodide (BMI), and LY225910 (Tocris, Ellisville, MO). Putative mitral cell somata were targeted for recordings based on their large diameter and location in a narrow band between the internal and external plexiform layers (mitral cell body layer). For brevity, when describing results we refer to the recorded population as ‘mitral cells’, with the understanding that we may have included a minority of internal tufted cells (or displaced mitral cells) which have similar morphology and reside in the deep EPL close to the mitral cell body layer [46], [47]. Patch clamp recordings were made using pipettes (5–20 MΩ) filled with (mM): 112 K-MeSO_4_, 10 K-glutamate, 26 K-HEPES, 0.2 K-EGTA, 2 Mg-ATP, 0.3 Na-GTP, 1 MgCl_2_, 4 Na_2_-phosphocreatine, 6.5 biocytin HCl, 0.2 Alexa Fluor 594, pH 7.2. Current or voltage signals were detected by an Axopatch 1D amplifier (Molecular Devices, Sunnyvale, California), conditioned with a 1 kHz 4-pole Bessel filter and digitized (sample interval 250 µs) by a HEKA ITC-18 interface (ALA Scientific Instruments) controlled by WinEDR V3.1.7 (University of Strathclyde, UK). Records were acquired continuously for up to 2000 s. We recorded CCK responses either as cell-attached action currents, or as changes in whole-cell current or voltage. Whole-cell input resistance was routinely monitored by hyperpolarizing voltage test pulses (−10 mV) delivered before and after record acquisitions. We only accepted cells with input resistances >100 MΩ. Morphology of cells recorded in whole-cell mode was determined either by *in vitro* fluorescence microscopy (filters: excitation 560 nm, dichroic 590 nm, emission 610 nm; Omega Optical, Brattleboro, Vermont) after loading cells with Alexa Fluor 594 (Life Technologies, Grand Island, New York), or by *post hoc* biocytin staining of neurons after slices were fixed overnight in phosphate buffered saline (PBS) with 2% glutaraldehyde (Vectastain Elite ABC kit, VIP peroxidase substrate kit; Vector Laboratories). Stained cells were viewed with a Nikon Microphot microscope.

### Data Analysis

We used MiniAnalysis software (Synaptosoft, Decatur, Georgia) to detect action currents or action potentials (‘spikes’), and excitatory/inhibitory postsynaptic potentials (EPSPs/IPSPs) or currents (EPSCs/IPSCs), and the timing data for these events was further analyzed in Origin 7.0 (OriginLab, Northampton, Massachusetts). Spike or EPSC/IPSC responses to CCK stimulation were quantified by computing time-dependent, sliding window event rates [48], *r*(*t*), with rectangular window of width 50 s. The mean and standard deviation (m*_r_*, sd*_r_*) of the rate function was calculated in a control period 100–200 s prior to the stimulus, and the criterion for an excitatory or suppressive response was if *r*(*t*) deviated above or below 99% confidence limits (m*_r_* ±3× sd*_r_*) respectively. The latencies of excitatory or suppressive responses were taken at the first *r*(*t*) crossings of these limits, and their durations as the interval between first and second crossings. Mean normalized responses over many cells were computed as the average of standardized rate functions obtained by subtracting a mean pre-stimulus rate and normalizing to maximum magnitude of the rate change. To reduce the contribution of random fluctuations in cell activity, we rejected unstable recordings in which large excursions in spike rate occurred in a 200 s prestimulus period. Our recording technique was able to acquire stable baseline activities in unstimulated control cells over at least 1200 s, sufficient time to capture most CCK responses. Slow changes in whole-cell currents or membrane potentials associated with CCK responses were tracked either by digital filtering (2-pole Bessel, 0.10 Hz), or by extracting baseline values from postsynaptic events detected by MiniAnalysis. In a minority of cells, small, steady drifts in prestimulus baseline were estimated by least squares linear fitting, and traces were corrected by trend subtraction. Mann-Whitney U tests [49] were applied to evaluate differences in response latencies and durations between groups of cells under control conditions and with synaptic blockers. Fractions of different classes of spiking response in drug treatment groups were compared to control groups by computing cumulative probabilities from corresponding trials of a Binomial distribution [49], taking control fractions as estimators of response probability for the null hypothesis (‘binomial test’). Summary data are expressed as mean ± SEM (standard error of the mean).

### Immunohistochemistry

Mice (P28) were euthanized by halothane overdose and olfactory bulbs were removed and fixed in 4% paraformaldehyde with PBS (mM: 138 NaCl, 2.7 KCl, 10 Na_2_HPO_4_, 2 KH_2_PO_4_; pH 7.4), for 20 h at 4°C, dehydrated in serial ethanol dilutions, Histo-Clear (National Diagnostics, Atlanta, Georgia), and mounted in paraplast. Bulbs were cut into 6–10 µm thick horizontal sections on a cryostat and paraffin sections were dried at 56°C for 15 min, then rehydrated, and washed 1×10 min in 10 mM PBS, then washed in citrate buffer (10 mM citric acid, 0.05% Tween 20, pH 6.0). Nonspecific antibody binding was blocked by 10% serum, 0.3% Triton X-100, 3% bovine serum albumin overnight. Sections were incubated in primary antibody in a humidified chamber (2 d, 4°C), then in secondary antibody conjugated with Alexa Fluor 488 or Alexa Fluor 633 (Life Technologies, Grand Island, New York) (1 h, 23°C). Sections were then washed in PBS and mounted in Vectorshield medium or Vectorshield with DAPI (4′,6 diamidino-2-phenylindole) (Vector Laboratories, Burlingame, California). The following antibodies and dilutions were used: anti-CCK_B_ 1∶200 (NBP1-00744, Novus Biological, Littleton, Colorado); B2, 1∶100 (gift from L. Mercer & P. Beart, University of Melbourne, Parkville, Victoria); anti-Tbx21, 1∶100 (NBP1-43299, mouse monoclonal, Novus Biological); and anti-CCK-8S, 1∶40 (#9303, CURE Digestive Diseases Center, UCLA, Los Angeles, California). Images of antibody labeled sections were acquired using a Leica TCS SP2 Spectral Confocal Microscope (Leica Microsystems, Buffalo Grove, Illinois). Profiles of CCK_B_-immunopositive somata in the mitral cell and glomerular/external plexiform layers were traced in Paint Shop Pro 9, and their maximum (Feret’s) diameters computed with ImageJ 1.45 s software [50], without application of tissue shrinkage correction.

### Ethics Statement

All experiments strictly followed the guidelines for animal care, handling and euthanasia set by the U.S. Public Health Service. Protocols were reviewed and approved by the Monell Chemical Senses Center Institutional Animal Care and Use Committee (Permit number: 1039).

## Results

### Excitation and Suppression of Mitral Cell Spiking Activity by CCK-8S

**Figure 1:**
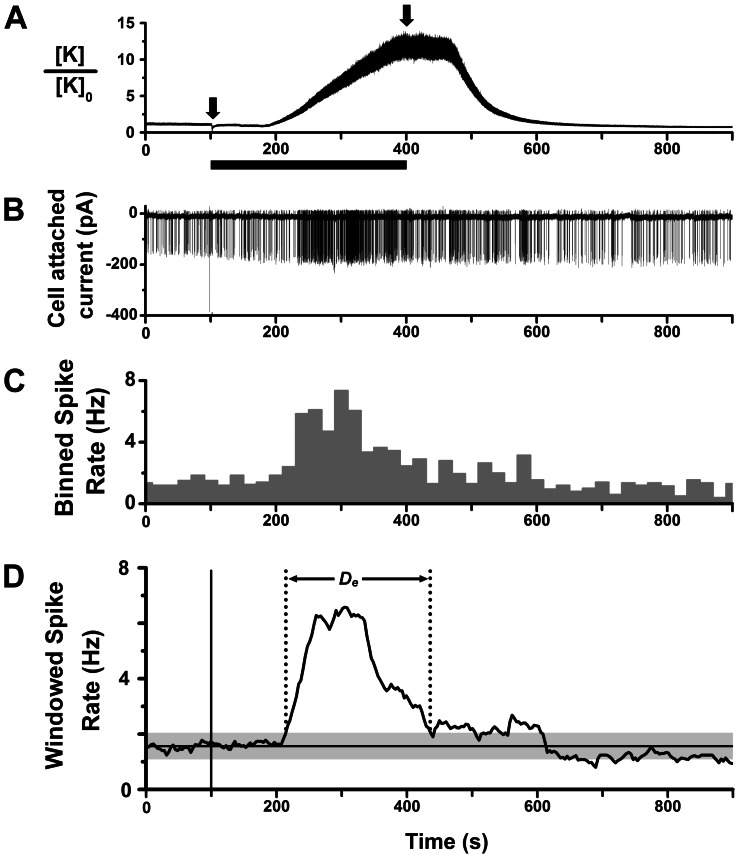
Cell-attached recording of mitral cell spike excitation by CCK-8S. **A.** Calibrated timing of agonist application, derived from measurement of K^+^ junction current of micropipette in olfactory bulb slice. Valve actuation at 100 s (left arrow) switched aCSF flow to high K^+^ perfusion, and washout was subsequently initiated at 400 s (right arrow). Black bar indicates time period of switched perfusion. Ordinate is the relative change in K^+^ concentration, estimated from shift in junction current at room temperature, ΔI [pA], as: [K]_0_ exp(0.004 ΔI), for 100 MΩ pipette. With a flow rate of 5 ml/min, the time lag in junction current shift was ∼80 s. Perfusion for 300 s was minimum time to attain final applied agonist concentration in the bath. **B.** Excitatory spike response of a mitral cell to bath perfusion of 1 µM CCK-8S, applied with time course shown in **A** (cell-attached patch recording of action currents, slice from CD-1 mouse). **C.** Histogram of spike rate over time (counts in 10 s bins) for the response in **B. D.** Plot of windowed spike rate vs. time, computed from spike times for response in **B**, with 50 s sliding window. Vertical line marks timing of CCK-8S perfusion switch. Horizontal line marks the mean pre-stimulus spike rate; gray band is 99% confidence limit (3× sd*_r_*) around the mean. Calculated duration of excitatory spike response (*D*_e_) is indicated.

**Figure 2:**
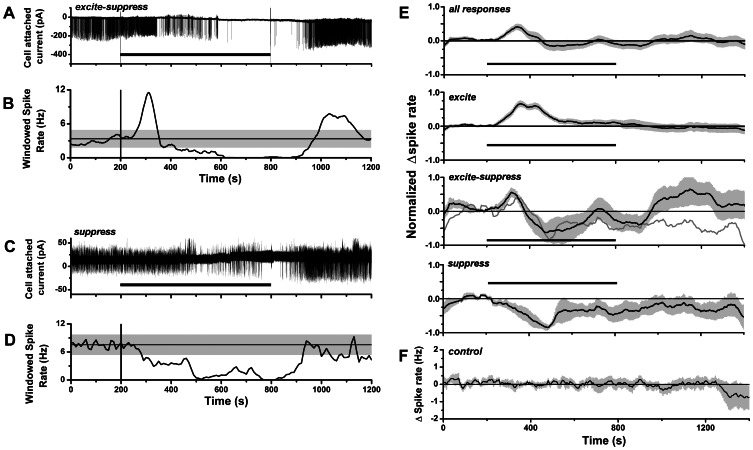
CCK-8S excitatory and suppressive spike responses in mitral cells. **A.** Action current record of mitral cell mixed excitatory-suppressive spike response to 1 µM CCK-8S perfusion (600 s, black bar), showing initial transient elevated firing phase followed by later suppression of spiking. **B.** Plot of windowed spike rate vs. time for response in **A.** **C.** Action current record of mitral cell suppressive spike response to 1 µM CCK-8S perfusion (600 s, black bar). **D.** Plot of windowed spike rate vs. time for response in **D.** In **B** and **D**, vertical lines indicate initial switching times for CCK perfusion, horizontal lines and gray bands indicate m*_r_* ±3× sd*_r_* limits for pre-stimulus spike rates. **E.** Mean normalized changes in spike rate of mitral cell responses to CCK. Mean rate plots are shown for: *top panel*: all classes of response (*n* = 37 cells); *second panel*: excitatory responses (*n* = 21 cells); *third panel*: biphasic excitatory-suppressive responses (*n* = 10 cells); *bottom panel*: suppressive responses (*n* = 6 cells). Mean curves were calculated from windowed spike rates offset relative to basal spike rates and normalized to the peak of excitation (for excitatory and excitatory-suppressive responses) or suppression (for suppressive responses). Gray curve in third panel is linear combination of mean excitatory and suppressive responses for least squares fit to biphasic response over 220–540 s period. **F.** Mean spike rate (baseline subtracted) for control mitral cells without CCK stimulation (*n* = 10). Records averaged were selected based on the same criterion applied to screen for acceptable records from CCK stimulus experiments, i.e. baseline spike rate was stable over at least an initial 200 s period. The plot shows that stable spike rates could be obtained for up to 1200 s, given stable rates over initial 200 s. In **E** and **F**: black curve = mean rate; gray band = SEM; horizontal black bar = CCK-8S perfusion switch (aligned to 200 s, maximum duration of CCK perfusion was 600 s).

We surveyed the effects of CCK on mitral cell spike activity in olfactory bulb slices from CD-1 mice by cell-attached patch clamp recording of action currents. This method facilitated stable, long term recordings (∼30–60 min), and avoided potential wash-out of CCK-linked G protein-coupled second messenger pathways [38]. For stimulation, we switched the extracellular perfusion medium to a solution containing 300 nM or 1 µM CCK-8S, for 300 s or longer, to allow time for peptide to attain a plateau concentration in the slice (Fig. 1A). Fig. 1B shows that under these conditions CCK-8S was able to elicit a robust increase in the spiking frequency of mitral cells lasting several minutes. Analysis of binned or windowed spike rates revealed a single excitatory response peak with maximal rate several-fold higher than the basal firing rate (Figs. 1C–D). In other cells, CCK-8S elicited a biphasic response composed of an early excitatory phase followed by an extended period of suppressed spike firing at rates below prestimulus rate (Figs. 2A–B). A third category of response was suppression of spiking without an initial excitatory phase (Figs. 2C–D). We never observed the reverse order of response, i.e. initial spike suppression followed by excitation. Of 42 recorded mitral cells, spike excitation was seen in 21 cells (50%), excitation-suppression in 10 cells (24%), and suppression in 6 cells (14%); 5 cells (12%) did not have a significant response. The excited cells included 4 silent cells that responded to CCK-8S by initiating spike activity.

Statistics for latencies, durations and magnitudes of the three classes of spike response are summarized in Table 1. A frequently observed property of excitatory responses was that after reaching a peak, the rate began to decline back to basal level even before initiation of washout of CCK-8S from the bath (17/21 cells), and total response duration was usually shorter than CCK perfusion time (18/21 cells) (Fig. 1). Thus, excitatory response termination was determined not by stimulus removal, but by physiological mechanisms. We observed a late rebound phase of elevated spiking following wash-out of CCK-8S in 4/21 cells with excitatory responses, 7/10 cells with mixed excitatory-suppressive responses (late hump in Fig. 2E), and 2/6 cells with suppressive responses. Overall, the strength of rebound was positively correlated with strength of initial excitation, as measured by ratios of peak to basal spike rates (Pearson’s r = 0.83, p = 2.6×10^–4^, *n* = 17, including BMI and CCK-4 data given below).

**Table 1:** Parameters characterizing mitral spike responses to CCK receptor agonists.

		Excitation			Suppression		
	*n*	latency (s)	duration (s)	rate delta (Hz)	latency (s)	duration (s)	rate delta (Hz)
CCK-8S (excite)	21	88.3±14.0	282.4±40.0	4.33±0.73	–	–	–
CCK-8S (excite-suppress)	10	131.6±22.7	188.2±54.0	5.48±1.34	348.2±61.6	298.7±57.6	–3.75±0.52
CCK-8S (suppress)	6	–	–	–	126.3±39.5	346.7±86.4	–6.58±1.37
CCK-8S+dCK/NBQX	5	96.2±12.66	257.0±127.3	2.52±1.16	–	–	–
CCK-8S+BMI	5	52.0±12.9	520.2±214.9	12.33±2.97	–	–	–
CCK-4 (excite)	4	78.3±22.7	315.0±99.5	5.82±1.40	–	–	–
CCK-4 (excite-suppress)	2	55.5±35.5	103.5±43.5	3.53±0.37	169±9	223±25	–1.22±0.20
CCK-4 (inhibit)	1	–	–	–	322	405	–1.64

**Figure 3:**
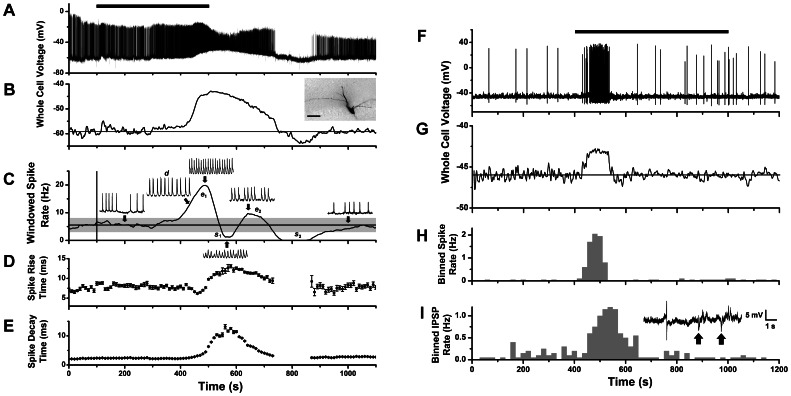
Whole-cell recording of mitral cell voltage response to CCK-8S stimulation. **A.** Whole-cell current clamp recording of membrane potential change of a mitral cell during perfusion with 1 µM CCK-8S (bar indicates 400 s period of switched perfusion) showing elevated firing correlated with a slow depolarizing potential of ∼16 mV amplitude. **B.** Slow depolarizing response revealed by applying low pass filter (0.10 Hz) to trace in **A.** Inset image: biocytin stain of recorded cell showing amputated apical dendrite (scale bar: 50 µm). **C.** Plot of windowed spike rate vs. time for response in **A**, showing several phases of spike excitation and suppression (*e*_1_, *s*_1_, *e*_2_, *s*_2_). All spike events were counted, including those with reduced amplitude (but doublets only once). The first suppressive phase (*s*_1_) correlated with a period of spike inactivation at depolarization peak, the second (*s*_2_) with a post-stimulus hyperpolarization. Inset traces: 1 s expanded segments of voltage record at different times (arrows): basal spike rate (200 s), rising phase (430 s), first peak (500 s), first minimum (570 s), second peak (650) and recovery (1000 s). **D–E.** Running average plots of spike rise times (**D**) decay times (**E**) for the response in **A** (mean ± standard deviation, in 10 s bins). **F.** Whole-cell current clamp recording of membrane potential from a second mitral cell with a spiking response to 1 µM CCK-8S perfusion (horizontal bar). **G.** Slow depolarizing response revealed by applying a low pass filter (0.10 Hz) to trace in **F.** **H.** Histogram of spike counts over time (20 s bins) for the trace in **F.** **I.** Histogram of IPSP counts over time (20 s bins) for the trace in **F.** Inset: expanded trace from **F** showing action potential and IPSPs (arrows) occurring during the CCK response.

First three rows list descriptive parameters under control conditions for three classes of CCK-8S spike response: excitatory, mixed excitatory-suppressive, and suppressive (data pooled for 300 nM and 1 µM CCK-8S; there was no significant difference between the two concentrations). For multiphasic responses caused by spike inactivation (e.g. Fig. 3C), duration of excitation was calculated using the end of the second excitatory peak. Next two rows list parameters of spike responses to 1 µM CCK-8S recorded in the presence of blockers of fast synaptic transmission. Last three rows list parameters of responses to 1 µM CCK-4, for 3 spike response classes: excitatory, mixed excitatory-suppressive, and suppressive. Values are expressed as mean ± SEM. Latencies were measured from time of CCK perfusion switch (including ∼50–80 s perfusion lag) to time of deviation from mean basal spike rate (p<0.01), and durations were computed from time points when responses deviated above or below (p<0.01) mean basal spike rate. Rate delta values for CCK responses are peak increments in spike rate relative to basal spike rate (positive for excitatory, negative for suppressive responses).

Fig. 2E shows mean time courses of responses plotted as the peak-normalized spike rate function averaged over all cells (upper trace), and separate mean rate functions for each response category (lower 3 traces). From these plots we see that: (i) the duration of the mean response of purely excitatory responses was longer than the duration of the mean excitatory phase of biphasic excitatory-suppressive responses; and (ii) the mean suppressive response peaked later than the mean excitatory response. We could approximate the mean time course of biphasic responses by a linear combination of mean excitatory and inhibitory responses. This suggested that in some cases biphasic responses might be produced by a superposition of independent excitatory and suppressive mechanisms.

### Electrical Basis of Spike Excitation and Suppression

We obtained whole-cell recordings from mitral cells to measure transmembrane currents and voltages underlying CCK-induced spike excitation and suppression. Under current clamp, we found that increased spike activity caused by CCK-8S was associated with a slow depolarizing potential (Fig. 3). Figs. 3A–E show data from a cell with an excitatory-suppressive spike response. The cell showed robust basal spike activity that was further elevated by a slow CCK-induced depolarization 16 mV above resting potential (excitation phase *e*_1_). Immediately after the peak of depolarization there was a transient reduction in firing rate (suppression phase *s*_1_), during which action potentials had curtailed amplitudes and longer rise times (Figs. 3C–D). These changes are consistent with the depolarization causing an inactivation of voltage-gated conductances underlying action potentials. During the rising phase of depolarization, action potentials were split into spike doublets (*d*) by a slower spike component that increased apparent spike decay times (Figs. 3C, E). This phenomenon is seen in other neurons and has been attributed to electrogenic calcium potentials [51]. Spike amplitude and spike frequency partially recovered from inactivation during the repolarization phase of the slow potential (excitation *e*_2_), and firing was then suppressed by a period of hyperpolarization (s_2_). Thus, CCK may suppress mitral cell firing either by spike inactivation following strong depolarization, or by hyperpolarization.

Figs. 3F–I show data from another cell with a CCK response classified as purely excitatory. In this cell, basal spike activity was much lower and was transiently elevated by a smaller CCK-induced depolarization (2.9 mV) that did not cause spike inactivation. In this case, depolarization was initiated with a shorter latency and adapted quickly, yielding a monophasic spike excitation (Fig. 3H). Although there was no significant spike suppression because of the low basal firing rate, inhibitory post synaptic potentials (IPSPs) were detectable as brief hyperpolarizing events that increased in frequency with a slight lag behind spike excitation (Fig. 3I). Thus, CCK could recruit inhibitory synaptic input from local interneurons while exciting the recorded mitral cell.

We detected a monophasic depolarizing potential in *n* = 7 mitral cells stimulated by CCK-8S, with amplitudes 1–25 mV (mean ± SEM, 7.88±3.80 mV), rise times 24–152 s (83±22 s) (in 6/7 cells with monostable membrane potential), and durations 121–505 s (292±78 s) (in 6/7 cells that returned to baseline). Mean duration was not significantly different from that of excitatory spike responses in cell-attached recordings (Table 1) (p = 0.97, Mann-Whitney test), which is consistent with the depolarization as the source of spike excitation. Indeed, each depolarizing potential was accompanied by increased frequency of action potentials. In 5 cells, the depolarization and attendant spiking terminated well before starting CCK wash-out, without spike inactivation or hyperpolarization. This indicated that the depolarizing potential activated by CCK was capable of physiological adaptation.

**Figure 4:**
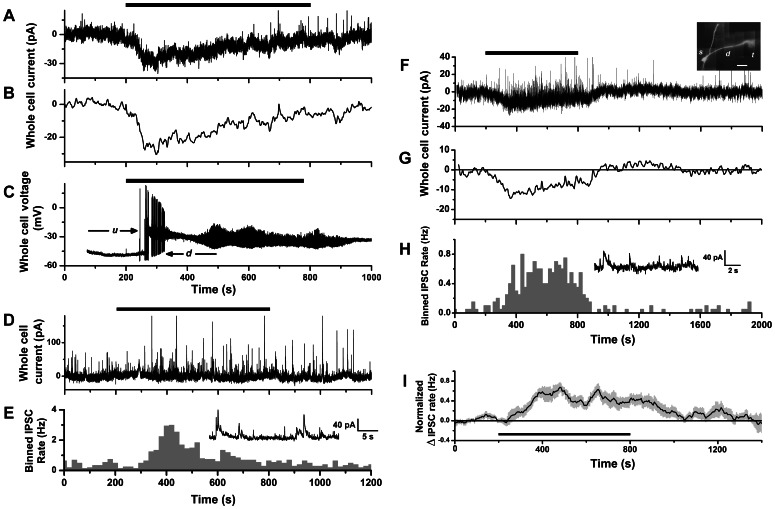
Whole-cell recording of mitral cell currents response to CCK-8S stimulation. **A.** Whole-cell voltage-clamp recording of membrane current of a mitral cell during perfusion with 1 µM CCK-8S (horizontal bar), showing slow inward current response. **B.** Time course of slow inward current revealed by applying a low pass filter (0.10 Hz) to trace in **A.** **C.** Whole-cell current-clamp recording from same cell as in **A**, showing voltage response to reapplication of 1 µM CCK-8S (horizontal bar). Depolarization caused the cell to switch between a lower voltage down-state (*d*) with action potential firing, and a high voltage up-state (*u*) with fast membrane potential oscillations, which are known properties of mitral cells [80]–[82]. **D.** Whole-cell voltage clamp recording from a second mitral cell during perfusion with 1 µM CCK-8S (horizontal bar), showing lack of slow inward current and increase in IPSC activity. **E.** Histogram of IPSC counts over time (20 s bins) for the trace in **F.** Inset: expanded segment from **D** showing IPSCs occurring during CCK stimulation. **F.** Whole-cell voltage clamp recording from a third mitral cell during perfusion with 1 µM CCK-8S (horizontal bar), showing both a slow inward current and an increase in IPSC activity. Inset: in vitro fluorescence image of cell loaded with Alexa 594 to reveal soma (*s*), apical dendrite (*d*) and glomerular tuft (*t*). Scale bar: 50 µm. **G.** Time course of slow inward current revealed by applying a low pass filter (0.10 Hz) to trace in **F.** **H.** Histogram of IPSC counts over time (20 s bins) for trace in **F.** Inset: expanded segment from **F** showing IPSCs occurring during CCK stimulation. **I.** Mean normalized change in IPSC rate of mitral cell voltage clamp responses to CCK-8S (*n* = 8 cells, calculated as described in Figs. 2E–F).

Under voltage clamp, a slow inward current was elicited in response to CCK-8S stimulation. The time course of the current was monophasic with an early peak and slow decay back to baseline in the continued presence of agonist (Figs. 4A–B). In 4 cells, we recorded a CCK-induced inward current under voltage clamp, washed out CCK, and then observed a depolarization and spiking response after a repeated CCK stimulation of the same cell under current clamp. This showed directly that the inward current was responsible for excitatory spike responses. In two such cells, the initial increase in spike rate was followed by an extended silent period, when depolarization was sufficiently large to prevent further action potential discharge (Fig. 4C). This was further evidence that the inward current itself could cause excitatory-suppressive responses by spike inactivation, as illustrated in Fig. 3A. We detected slow inward currents in *n* = 9 mitral cells stimulated by CCK-8S, with amplitudes 12.5–37 pA (mean ± SEM, 21.44±2.88 pA), rise times 80–286 s (170±25 s), and durations 302–830 s (630±68 s) (in 8/9 cells that returned to baseline). Mean duration was not significantly different from total durations of excitatory-suppressive spike responses in cell-attached recordings (487±58 s, *n* = 10, p = 0.11, Mann-Whitney test).

In some cells we observed a significant increase in the frequency of inhibitory post synaptic potentials (IPSCs), appearing as a barrage of transient outward currents after switching to CCK perfusion. This could occur either in the absence (Figs. 4D–E) or presence (Figs. 4F–H) of the slow inward current response. An IPSC response was seen in 3 cells that lacked an inward current, and among 8 cells with inward currents an IPSC response was present in 5/8, absent in 3/8. Thus, recruitment by CCK of inhibitory synaptic input to mitral cells can occur independently of an inward current response. The IPSC responses had durations of 280–892 s, (mean ± SEM, 569±67 s), which was not significantly different from total durations of excitatory-suppressive spike responses (p = 0.45, Mann-Whitney test). In Fig. 4I, we plot the mean peak-normalized IPSC rate function averaged over 8 cells. This time course compares favorably with the mean time course of suppressive spike responses (Fig. 2E). In summary, our whole-cell recordings revealed a CCK-activated slow inward current that can generate excitatory spike responses, and that spike suppression could arise both from spike inactivation by sustained depolarization, and potentially from inhibitory synaptic input recruited by CCK.

Whole-cell dialysis with fluorescent tracer or biocytin allowed us to visualize dendrites of recorded cells (Fig. 4F, inset). The apical dendritic tuft was amputated in 4 cells recorded under voltage clamp, 2/4 of which displayed a slow inward current, and 3/4 an IPSC response. In addition, 2 cells recorded under current clamp lacked a tuft (Fig. 3B, inset) but responded to CCK with slow depolarization and increased rate of spiking. Hence, the apical tuft was not required for either the slow depolarizing response or the recruitment of synaptic inhibition.

### Synaptic Contributions to Spike Excitation and Suppression

**Figure 5:**
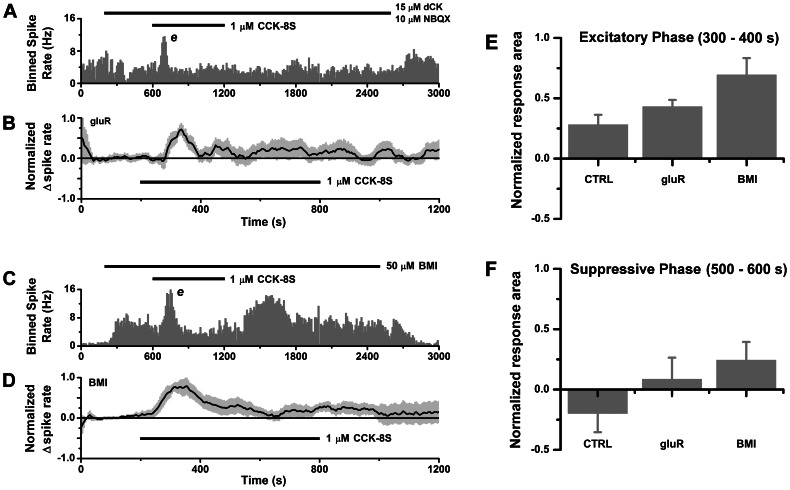
Synaptic contributions to spike excitation and suppression. **A–B.** Block of AMPA/kainate and NMDA receptors eliminates suppressive response leaving excitatory response intact. **A.** Histogram of spike counts over time (10 s bins) for cell-attached spike recording from a mitral cell during perfusion with NMDA and AMPA/kainate antagonists 15 µM dichlorokynurenate (dCK) +10 µM NBQX (upper horizontal bar, 200–2600 s), and dCK+NBQX +1 µM CCK-8S (lower horizontal bar, 600–1200 s). Block of tonic glutamatergic excitation by the antagonists caused a reduction in basal spike rate, which recovered after washout. In the presence of antagonists, addition of CCK-8S evoked a transient rise in spike rate with no suppressive phase. **B.** Mean normalized change in spike rate for excitatory responses to 1 µM CCK-8S under ionotropic glutamate receptor (gluR) block (*n* = 5 cells). **C–D.** Block of GABA_A_ receptors eliminates the suppressive response leaving excitatory response intact. **C.** Histogram of spike counts over time (10 s bins) for cell-attached spike recording from a mitral cell during perfusion with 50 µM bicuculline methiodide (BMI) (upper horizontal bar, 200–2500 s) and BMI +1 µM CCK-8S (lower horizontal bar, 600–1200 s). Block of tonic GABAergic inhibition by BMI caused an elevation in basal spike rate, which was restored to baseline after washout. In the presence of BMI, addition of CCK-8S evoked a transient increase in spike rate, with adapted phase and rebound excitation after removal of CCK. **D.** Mean normalized change in spike rate for excitatory responses in BMI (*n* = 5 cells). **E–F.** Comparison of mean areas under normalized spike rate plots for time periods (300–400 s, and 500–600 s after perfusion switch) corresponding to excitatory (**E**) and suppressive (**F**) phases of CCK responses, respectively. CTRL: control; gluR: with NBQX and dCK; BMI, with bicuculline methiodide. Data averaged over all cells including non-responders. Error bars: SEM.

The spike excitation of mitral cells by CCK could be mediated either directly by activation of postsynaptic CCK receptors on mitral cells, or indirectly by excitation of other cells that release glutamate onto mitral cells. To distinguish these possibilities, we applied CCK-8S while blocking ionotropic glutamate receptors with NBQX or CNQX (competitive antagonists of AMPA/kainate receptors), and dichlorokynurenate (dCK) (non-competitive antagonist of NMDA receptors). In 5 of 8 tested cells, we could still record excitatory spike responses (3 cells non-responsive) (Figs. 5A, 5B). Between-groups comparison showed that this fraction was not significantly different from control excitation frequency in the absence of antagonists (30/41 = 73.2%) (p = 0.37, binomial test), and there was no significant difference in either response latency (p = 0.68), duration (p = 0.43), or peak increase in spike frequency (p = 0.15) (Mann-Whitney test) (Table. 1). Thus, mitral cell excitation by CCK does not require participation of ionotropic glutamate receptors. We did not observe any spike responses of the excitatory-suppressive or purely suppressive types under glutamate receptor block (0/8 cells), a significant deviation from the incidence of suppression in control data (16/42 = 38%; p = 0.022). This is consistent with a dependence of suppressive responses on glutamatergic excitation of other neurons.

The ability of CCK to recruit IPSC input to mitral cells suggested that GABAergic inhibition may contribute to spike suppression. To test this, we applied CCK-8S while blocking GABA_A_ receptors with bicuculline methiodide (BMI), and found that 5/8 cells tested responded with excitation only (no suppressive phase) (Figs. 5C, 5D), and 2/8 cells were unresponsive. One remaining cell responded with biphasic excitation-suppression, but the suppressive phase was associated with increased spike rise and decay times, implicating spike inactivation as the cause (similar to the cell in Figs. 3C–E). Between-groups comparison showed that the lack of spike suppression in BMI deviated significantly from the incidence of suppression in control data (p = 0.035, *n* = 7). An excitatory phase was still present in BMI, and was similar to control responses in latency (p = 0.18) and duration (p = 0.75) (Mann-Whitney test, Table 1). The peak increase in spike frequency in BMI was higher than control (p = 0.013, Mann-Whitney test, Table. 1). This would be expected if onset of GABAergic inhibition overlapped and reduced the magnitude of the excitatory phase. In all cases, the excitatory response in BMI terminated and basal spike rate was restored in the continued presence of CCK (Fig. 5B). Thus, adaptation, spike inactivation and synaptic inhibition may all be able to influence the time course of recovery from CCK excitation. We also observed rebound excitation in 4/5 excitatory responses recorded in BMI (Fig. 5C). In Figs. 5E–F we compare areas under the curve at different times for normalized spike rate functions to illustrate selective effects of synaptic blockers on excitation and suppression. The earlier excitatory phase was not changed much relative to control when glutamate receptors were blocked, but was significantly boosted when GABA_A_ receptors were blocked (Fig. 5E). However, the later suppressive phase (negative area in the control) became more positive when both glutamate and GABA_A_-mediated transmission were blocked (Fig. 5F).

### Roles of CCK Receptor Subtypes in Spike Excitation and Suppression

**Figure 6:**
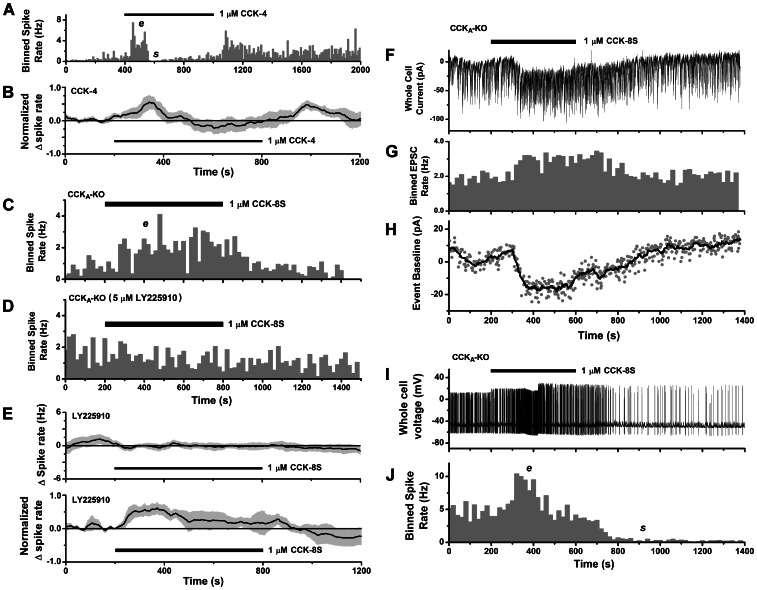
Contribution of CCK_B_ receptors to mitral cell excitation. **A–B.** CCK_B_-selective agonist CCK-4 evokes excitatory and suppressive spike responses. **A.** Histogram of spike counts over time (10 s bins) for a cell-attached spike recording from a mitral cell (CD-1 mouse) stimulated by perfusion with CCK-4 (horizontal bar). CCK-4 evoked a transient excitatory phase (*e*), followed by spike suppression (*s*), and after removal of stimulus there was a rebound excitation. **B.** Mean normalized spike rate for all mitral cell responses evoked by CCK-4 in CD-1 mice (*n* = 7 cells, 4 excitatory, 2 mixed excitatory-suppressive, 1 suppressive). **C–D.** Stimulation by 1 µM CCK-8S causes spike excitation in CCK_A_ knockout mice. **C.** Histogram of spike counts over time (20 s bins) for cell-attached spike recording from a mitral cell from a CCK_A_ knockout mouse, showing excitation (*e*) in response to CCK stimulus (horizontal bar). **D.** Histogram of spike counts from the same cell after washout and reapplication of 1 µM CCK-8S (horizontal bar) in the presence of a CCK_B_-selective antagonist (5 µM LY 225910) which blocked spike excitation. **E.** *Upper panel*: mean spike rate (baseline subtracted) for mitral cells unresponsive to CCK-8S in the presence of LY225910 (*n* = 9). *Lower panel*: mean normalized spike rate for excitatory spike responses evoked by CCK-8S in the presence of LY225910 (*n* = 3 cells). All data from CD-1 mice. **F–J.** Inward current underlies spike excitation in the CCK_A_ knockout mouse. **F.** Whole-cell voltage clamp recording from a mitral cell from a CCK_A_ knockout mouse stimulated with 1 µM CCK-8S (horizontal bar), showing both slow inward current and an increase in EPSC activity, including currents underlying long lasting depolarizations (LLDs) [55]. **G.** Histogram of EPSC counts over time (20 s bins) for the trace in **F**, showing excitatory effect of CCK. **H.** Time course of slow inward current response to CCK for the trace in **F**, estimated by plotting baseline values for detected EPSCs (gray circles). Black line: smoothed curve obtained by averaging window of 8 data points. **I.** Whole-cell current clamp recording from same cell as in **F**, showing strong spike excitation caused by reapplication of 1 µM CCK-8S (horizontal bar). **J.** Histogram of spike counts over time (20 s bins) for the voltage trace in **I**, showing periods of spike frequency excitation (*e*) and suppression (*s*).

Both CCK_A_ and CCK_B_ receptor subtypes have been detected in the olfactory bulb [41], [42], and either one could mediate modulatory actions of CCK on mitral cell activity. To assess their relative contributions, we first applied the tetrapeptide CCK-4 (1 µM), an agonist that selectively binds the CCK_B_ receptor with nearly 2 log units higher affinity than the CCK_A_ receptor [52]. Fig. 6A shows a typical mitral cell response with transient phases of spike excitation and suppression. In 9 spontaneously active cells, CCK-4 evoked spike excitation (4/9), excitation–suppression (2/9), suppression (1/9), or no response (2/9) (mean response time course shown in Fig. 6B). There was no significant difference from CCK-8S in the incidence of excitation (6/9 = 66.7%, p = 0.815) or suppression (3/9 = 33.3%, p = 0.533) (binomial tests), and in latency (p = 0.59) and duration (p = 0.95) of excitatory responses (Mann-Whitney tests) (Table 1). Rebound excitation also occurred in 4/5 cells with excitatory responses after wash-out of CCK-4 (e.g. Fig. 6A). Thus, stimulation of CCK_B_ receptors with CCK-4 elicited spike responses with properties similar to those of CCK-8S responses.

We also evaluated the contribution of CCK_B_ receptors by studying CCK responses in mitral cells from CCK_A_ knockout mice [53]. Figs. 6C–D show that 1 µM CCK-8S could evoke spike excitation in the CCK_A_ knockout, and that this response was abolished in the presence of a CCK_B_ receptor-selective competitive antagonist, 5 µM LY225910 [14], [54]. Transient spike excitations were observed in 2/5 cells tested in CCK_A_ knockouts, while 3/5 were unresponsive. Figs. 6F and 6H show activation of a slow inward current in a CCK_A_ knockout mitral cell. During this response, we also observed an elevation in the frequency of spontaneous EPSCs (Fig. 6G), including the large, relatively slow currents corresponding to long lasting depolarizations (LLDs) seen in mitral cells [55]. This showed that exogenous CCK was able to excite and accelerate the collective activity of intraglomerular mitral/tufted cell networks generating the LLD [56], [57]. Figs. 6I–J show that after removal of CCK, repeated stimulation of the same cell under current clamp evoked a transient increase in action potential discharge. Thus, CCK_B_ receptors can generate a slow inward current and mediate spike excitation.

**Figure 7:**
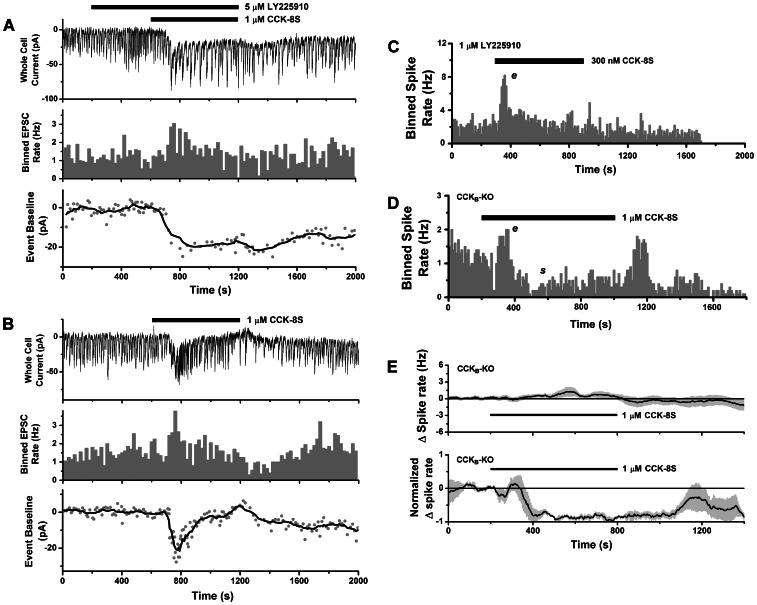
Contribution of CCK_A_ receptors to mitral cell excitation. **A–B.** CCK evokes an excitatory inward current when CCK_B_ receptors are blocked (compare to Figs. 6F–H). **A.** *Upper panel*: whole-cell voltage clamp recording from a mitral cell (CD-1 mouse) stimulated with 1 µM CCK-8S (lower horizontal bar), in the presence of 5 µM LY225910 (upper horizontal bar), a CCK_B_-selective antagonist. An inward current response was accompanied by increased EPSC activity (LLDs). *Middle panel*: histogram of EPSC counts over time (20 s bins) showing excitatory effect of CCK. *Lower panel*: time course of slow inward current response estimated by plotting baseline values for detected EPSCs (gray circles). Black line: smoothed curve obtained by averaging window of 8 data points. **B.** *Upper panel*: voltage clamp recording of inward current and EPSC from same cell as in **A**, stimulated with 1 µM CCK-8S (horizontal bar) after washout of LY225910, showing shorter inward current followed by outward current. *Middle panel*: histogram of EPSC counts over time (20 s bins) showing excitation followed by reduction in activity. *Lower panel*: Time course of slow inward/outward current response, estimated as in **A.** **C.** Histogram of spike counts over time (20 s bins) for cell-attached recording from a mitral cell (CD-1 mouse) showing excitation (*e*) in response to 300 nM CCK-8S (horizontal bar), in the presence of 1 µM LY225910 (pretreated slice). **D.** Histogram of spike counts over time (20 s bins) for cell-attached recording from a mitral cell from a CCK_B_ knockout mouse showing excitation (*e*) and suppression (*s*) in response to 1 µM CCK-8S (horizontal bar). In this cell, spike rate rebounded noticeably after CCK washout. **E.** *Upper panel*: mean spike rate (baseline subtracted) for mitral cells from CCK_B_ knockout mice unresponsive to CCK-8S (*n* = 13). *Lower panel*: mean normalized spike rate for excitatory and excitatory-suppressive spike responses in CCK_B_ knockout mice evoked by CCK-8S (*n* = 3).

Although the data from CCK_A_ knockout mice showed that CCK_B_ receptors were competent for generating an excitatory response, their expression in the olfactory bulb could be altered by pleiotropic effects [58]. We therefore also characterized the effects of CCK_B_ block by the antagonist LY225910 on spiking activity of mitral cells from wild-type (CD-1) mice. In the presence of LY225910 (1 µM or 5 µM), mitral cells did not respond to CCK-8S (300 nM or 1 µM) in 9/12 cells tested (Fig. 6E *upper panel*), while 3/12 cells responded with spike excitation (Figs. 6E *lower panel*, 7C). The fraction of non-responsive cells in LY225910 (75%) was significantly higher than control (12%). Taken together with results from selective agonist (CCK-4) experiments, this supports a role for CCK_B_ receptors in mediating mitral cell excitation in wild type mice. However, the persistence of excitation in some mitral cells in LY225910 also indicated involvement of CCK_A_ receptors. Fig. 7A shows that when CCK_B_ receptors are blocked by LY225910, application of CCK can still evoke a slow inward current, and increase the rate of EPSC activity in some mitral cells. In the presence of 5 µM LY225910, a slow inward current was evoked by 1 µM CCK-8S in 4/6 cells (12.6–34 pA, mean ± SEM 25.15±4.78 pA), while 2/6 were unresponsive. Thus, it appears that both CCK receptor subtypes can excite mitral cells.

To further examine the possible contribution of CCK_A_ receptors to spike excitation or suppression, we also recorded from mitral cells in CCK_B_ knockouts. Fig. 7D shows that CCK could evoke a excitatory and suppressive response in the CCK_B_ knockout. We did not detect modulation of spike activity by 1 µM CCK-8S in 13/17 cells from CCK_B_ knockouts (Fig. 7E, upper panel), while 4/17 cells responded with spike excitation (1/4), excitation-suppression (1/4), or suppression (2/4) (Fig. 7E, lower panel). The fraction of non-responders (76%) was significantly higher than in controls (12%, CD1 mice), and was consistent with the fraction seen when CCK_B_ receptors were blocked by LY225910.

### Localization of CCK_B_ Receptors on Mitral and Tufted Cells

**Figure 8:**
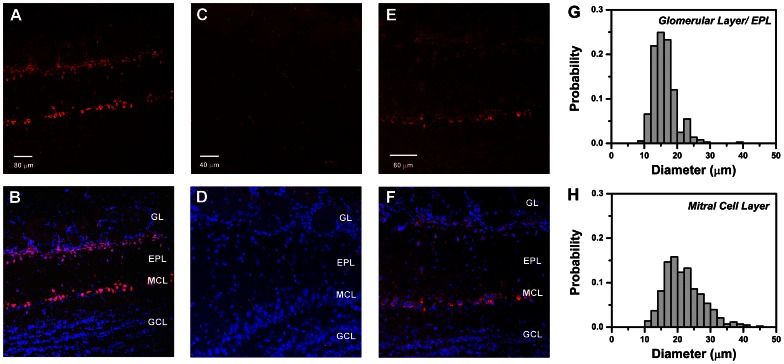
Cellular localization of CCK_B_ receptor immunoreactivity in mouse olfactory bulb. **A.** Confocal fluorescent image of anti-CCK_B_ receptor antibody binding to a horizontal section of an olfactory bulb from a CD-1 mouse, visualized with Alexa Fluor 633-conjugated secondary antibody (red). Strong immunoreactivity appears in a superficial zone including the inner margins of the glomerular layer (GL) and the distal part of the external plexiform layer (EPL), and in a deeper zone corresponding to the mitral cell body layer (MCL). Scale bar: 80 µm. **B.** Composite image showing overlay of CCK_B_ immunoreactivity in **A** (red) with fluorescent DAPI-stained nuclei (blue) to highlight the positions of cell bodies. Similar cellular distributions of immunoreactivity were obtained in 29 sections from 5 mice. **C.** Confocal fluorescent image (Alexa Fluor 633) of horizontal section of olfactory bulb from a 129-Cckbr^tm1Kpn^/J (CCK_B_ knockout) mouse, processed with the same antibody and protocol as in **A.** Scale bar: 40 µm. **D.** Composite image from the CCK_B_ knockout obtained by overlaying Alexa Fluor 633 fluorescence in **C** (red) with DAPI stained nuclei (blue). A similar absence of cell labeling was seen in 4 other sections of the bulb from the same mouse. **E.** Confocal fluorescent image of anti-CCK_B_ receptor binding to horizontal section from a CD-1 mouse, using B2 antibody of Mercer & Beart (2000) [42](1∶100 dilution). Scale bar: 80 µm. **F.** Composite image combining **E** (red) with fluorescent DAPI stained nuclei (blue). **G.** Distribution of cell body diameters in the GL and EPL that were immuno-positive for CCK_B_ receptor. **H.** Distribution of cell body diameters in the glomerular layer and EPL that were immuno-positive for CCK_B_ receptor. Histograms in **G**–**H** are normalized to total cell count. Note: Small non-nucleated fluorescent filaments visible in some sections are due to non-specific background staining of erythrocytes and appear in controls not treated with primary antibody. Abbreviations: GL, glomerular layer; EPL, external plexiform layer; MCL, mitral cell layer; GCL, granule cell layer.

The persistence of mitral cell excitation under synaptic block, and the recording of a slow inward current activated by CCK in mitral cells suggests that CCK receptors are likely to be expressed on mitral cells. To test this directly, we conducted immunocytochemical studies of CCK receptor expression in mouse olfactory bulb. We report results of experiments using antibodies targeting the CCK_B_ receptor subtype which is widely expressed throughout the brain. We screened commercially available antibodies and found that NBP1-00744 (Novus Biological), a rabbit polyclonal raised against a synthetic peptide around leucine 54 of human CCK_B_, yielded high contrast labeling of specific cell populations in mouse olfactory bulb (1∶200 dilution of 1.0 mg/ml stock). The typical pattern of immunofluorescence labeling by this antibody in CD-1 mice is illustrated in Figs. 8A–B. Strong fluorescence was observed in numerous cell bodies distributed across two main bands: (i) a broad band along the inner margin of the glomerular layer (GL) and distal part of the external plexiform layer (EPL); and (ii) a narrow band corresponding to the mitral cell body layer (MCL). Some isolated labeled cells were scattered in the middle and proximal parts of the EPL. However, there was a conspicuous absence of labeled granule cells, whose somata were clearly visualized by nuclear counterstain (Fig. 8B). Similar results were obtained from *n* = 29 sections, taken from 5 mice.

We confirmed that the observed labeling was specific for the CCK_B_ receptor by applying the same antibody and protocol to sections prepared from a CCK_B_ knockout mouse. The olfactory bulb of the knockout did not exhibit any obvious structural abnormalities and cells were organized into the same major layers as in wild type mice (i.e. glomerular, external plexiform, mitral cell, internal plexiform, and granule cell layers). In contrast to CD-1 mice, no significant antibody binding was detected in cells of any layer in bulb sections from the knockout (Fig. 8C–D; *n* = 5 sections from 1 mouse, processed in parallel with CD-1 sections). For independent confirmation of our results, we acquired and tested B2 antiserum raised against the conserved third intracellular loop domain of the CCK_B_ receptor, that was previously used to visualize expression of the receptor in rat brain [42]. Although the fluorescence contrast was weaker, the B2 antibody revealed the same staining pattern, with a majority of labeled cells located along a zone across the inner GL/distal EPL, and the MCL (Figs. 8E–F), but no labeling of granule cell bodies (*n* = 6 sections taken from 3 mice). We tested and rejected 8 other antisera that yielded low contrast, non-specific staining of entire bulb sections.

The sizes of CCK_B_-positive somata in the GL/EPL appeared to be smaller than those in the MCL. The difference was visualized in histograms of their diameter distributions (Figs. 8G–H). The mean ± SEM, and median diameter was: 16.16±0.18 µm, and 15.63 µm for GL/EPL somata (*n* = 365 cells); and 21.86±0.28 µm, and 20.94 µm for MCL somata (*n* = 430 cells). Thus, immunoreactive somata in GL/EPL were significantly smaller than those in MCL (Mann-Whitney test, p<0.001). The locations of, and size differences between these two groups of CCK_B_ positive cells are consistent with their identification as superficial tufted or juxtaglomerular cells, and mitral cells respectively [46].

**Figure 9:**
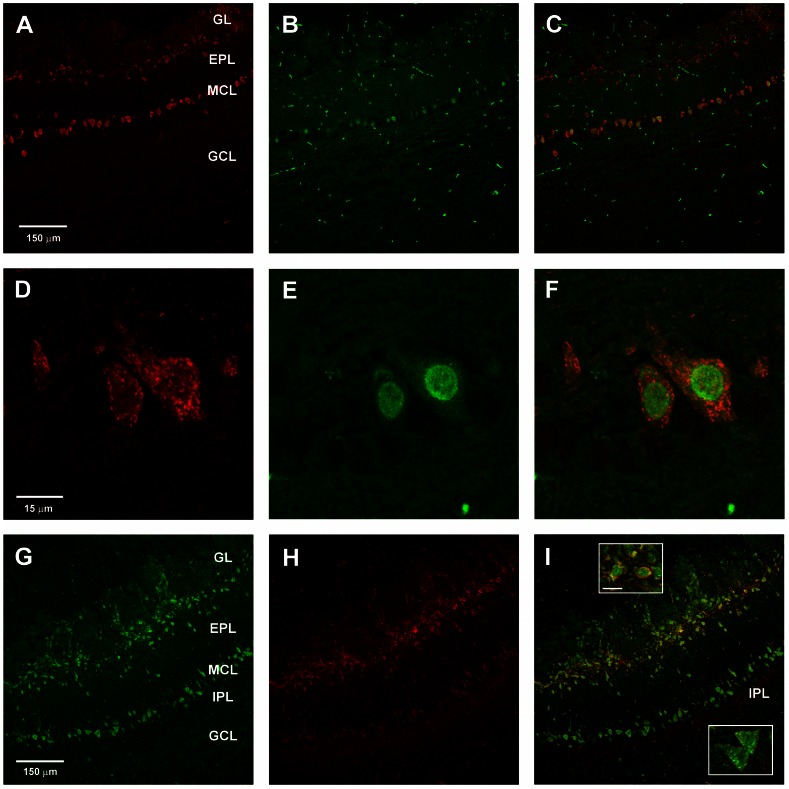
Colocalization of CCK_B_ receptor immunoreactivity in mouse olfactory bulb. **A–F.** Colocalization of immunoreactivity to CCK_B_ receptor and mitral/tufted cell nuclear marker Tbx21. **A–C.** Confocal fluorescent images of double labeled horizontal olfactory bulb section from a CD-1 mouse showing binding of antibody to CCK_B_ receptor (**A**) (red, Alexa Fluor 633), to Tbx21 (**B**) (green, Alexa Fluor 488), and the overlay of both images (**C**). Scale bar: 150 µm. **D–F.** Higher magnification view of mitral cell somata in the double labeled section, showing nuclear localization of Tbx21, and contrasting extranuclear distribution of CCK_B_. Scale bar: 15 µm. **G–I.** Confocal fluorescent images of double labeled horizontal olfactory bulb section from a CD-1 mouse showing binding of antibody to CCK_B_ receptor (**G**) (green, Alexa Fluor 488), to CCK-8S (**H**) (red, Alexa Fluor 633), and the overlay of both images (**I**). In the overlay, yellow labeling in the GL/EPL layer indicates cellular colocalization of receptor and peptide, whereas separated green and red bands in MCL and IPL indicates spatial separation of receptor and peptide. Scale bar: 150 µm. Abbreviations: see Figure 8. Insets in **I**: upper inset shows magnified view of cell somata in the superficial EPL (putative superficial tufted cells) with double labeling for peptide and CCK_B_ receptor; lower inset shows somata in mitral cell body layer (putative mitral cells) with single labeling for CCK_B_ receptor; scale bar for both in upper inset: 15 µm.

To further test whether CCK_B_ immunoreactivity was present in mitral cells, we next performed double immunofluorescence experiments to label both the receptor and a nuclear transcription factor, Tbx21, a T-box gene product that has been used as a marker for mitral and tufted cells [59]–[62]. We found that all cells in the MCL with nuclei positive for Tbx21, were also positive for CCK_B_ (*n* = 300 nuclei, from 7 sections, 2 CD-1 mice) (Figs. 9A–F). This demonstrated widespread expression of CCK_B_ receptors on mitral cell somata. Localization of CCK_B_ receptors in two separated layers, the GL/EPL and MCL, is similar to the two-tiered distribution of CCK-8S peptide immunoreactivity originally reported in somata superficial tufted cells, and in fibers of the internal plexiform layer (IPL) immediately below the mitral cell layer of rat olfactory bulb [20], [27]. Using an antibody against CCK-8S, we could also detect the peptide in cells in the distal EPL where superficial tufted cells reside, and in the IPL (Fig. 9H). Double immunostaining for both CCK_B_ and the peptide revealed cellular colocalization in a proportion of somata in the GL/EPL zone, where CCK-synthesizing superficial tufted cells are known to reside (Fig. 9I). However, in the MCL, localization of the receptor on mitral cell somata was separate from the peptide which was found in the IPL immediately below the MCL, as reported previously in other studies.

## Discussion

We found that mitral cells in olfactory bulb slices responded to exogenously delivered CCK either by spike excitation, suppression, or mixed (biphasic or polyphasic) excitation-suppression. Excitation always occurred first in mixed responses suggesting that this is the primary mode of action. Direct excitation of mitral cells by CCK was supported by the observation of slow depolarizing potentials and inward currents, and the persistence of excitatory spike responses in cells under block of glutamatergic synaptic transmission. With inhibitory transmission blocked, spike suppression was not observed. The results with synaptic blockers are explained most parsimoniously by a model in which CCK directly excites a population of mitral cells. Some of these mitral cells in turn excite local interneurons via glutamatergic transmission, which causes other mitral cells to receive GABAergic inhibition, as revealed by increased IPSC activity seen in voltage-clamp recordings. Another possibility is that CCK bypasses glutamatergic pathways and recruits GABAergic input to mitral cells by directly exciting interneurons [27]. Although we did not see spike suppression under glutamatergic block in the cells we tested, the negative finding does not rule out this hypothesis and more experiments are needed to test it. The persistence of excitatory responses in BMI does exclude a model in which CCK increased spiking in mitral cells by inhibiting interneurons and relieving tonic inhibition. The diversity of types of modulatory spike response to CCK might be a consequence of heterogeneity in mitral cells regarding CCK receptor expression, as well as variability in their intrinsic properties [63]–[65]. On the other hand, the colocalization of Tbx21 marker and CCK_B_ immunoreactivity does suggest widespread expression of this receptor in mitral/tufted cells. Variability in response is also expected for in vitro slice experiments in which exogenously applied agonist leads to parallel activation of many circuits throughout a slice, and this may be compounded if neurons suffer variable damage from slice cutting.

Our pharmacological experiments with CCK-4 and LY225910 indicated that both CCK_A_ and CCK_B_ receptors may participate in producing the excitatory response of mitral cells. Further evidence for a role of both receptor subtypes is the presence of CCK excitatory responses in both CCK_A_ and CCK_B_ knockout mice. A caveat of knockout experiments is the potential for compensatory effects altering normal expression levels of receptors [58]. However, we found a similar fraction of non-responsive cells when CCK_B_-mediated responses were excluded either by pharmacology or gene targeting, so compensation may not have major effects at the population level. It remains an open question whether CCK_A_ and CCK_B_ receptor subtypes are expressed in overlapping or exclusive subpopulations of mitral/tufted cells, and whether they couple to different signal transduction pathways in different cells. In a few whole-cell recordings, we detected signs of a post-excitatory phase of slow hyperpolarization or outward current not associated with an increase in inhibitory synaptic activity (Figs. 3B, 4G), which could indicate a postsynaptic mechanism for suppressive responses. For the cell in Figs. 7A–B, this outward current appeared to depend on CCK_B_ receptors. However, spike excitation and inward currents can also be mediated by CCK_B_ receptors (Fig. 6). Further studies are needed to determine if there is heterogeneity in CCK_A_/CCK_B_ receptor expression, or mode of action, among different mitral cells.

Our results suggest new roles for CCK signaling in intrabulbar association circuits. Previously it was theorized that CCK might be co-released alongside glutamate from superficial tufted cell terminals in the IPL, where it would bind to CCK receptors on granule cell apical dendrites and enhance their excitation, thus increasing inhibition of mitral cells [27]. Our finding that mitral cells are directly excited by CCK suggests a modified model with two parallel pathways converging on mitral cells. In the first pathway, glutamate from IPL terminals excites apical dendrites of granule cells, which inhibit isofunctional mitral cells. This disynaptic relay may be fast enough to operate within a sniff cycle to coordinate fine temporal coding of odors by output neurons from isofunctional pairs of glomeruli [66]–[68]. We leave open the possibility that granule cells may also be excited by CCK. Although we localized CCK_B_ receptors on mitral/tufted cells and not granule cells, we cannot rule out the expression of CCK_A_ receptors on granule cells. In the second pathway, released CCK diffuses up from the IPL to mitral cell bodies and excites them through CCK receptors. The spatial separation between CCK sources in the IPL and CCK receptor targets on mitral cells necessitates a diffuse paracrine mode of transmission similar to other peptidergic systems [69]. Mitral cell excitation by this pathway is expected to be much slower than mitral cell inhibition through the granule cell relay. The estimated diffusion coefficient of CCK-8S based on molecular weight [70] is ∼4.1×10^−6^ cm^2^s^−1^. From this we can calculate that it requires ∼3 s for liberated peptide to diffuse ∼50 µm from the IPL to the mitral cell layer to attain a concentration comparable to that in the vicinity of release sites. If CCK were released at nM concentrations in the range of CCK receptor affinity [71], diffusive transmission will be too slow for this pathway to participate in rapid computations of odor discrimination performed in one or a few short sniff cycles lasting only ∼360 ms in rodents [66], [72], [73]. However, CCK may cause slower, or tonic increases in mitral cell excitation when odorants are sampled by repeated sniffing over longer time periods, or are continuously present in the environment. We note that the adaptation and termination of the response during sustained CCK stimulation seen in our experiments might not occur if CCK activates receptors at lower nM range concentrations in vivo. We applied higher concentrations (300 nM–1 µM) to maximize the activation of receptors, to give the best chance of measuring small modulatory responses.

**Figure 10:**
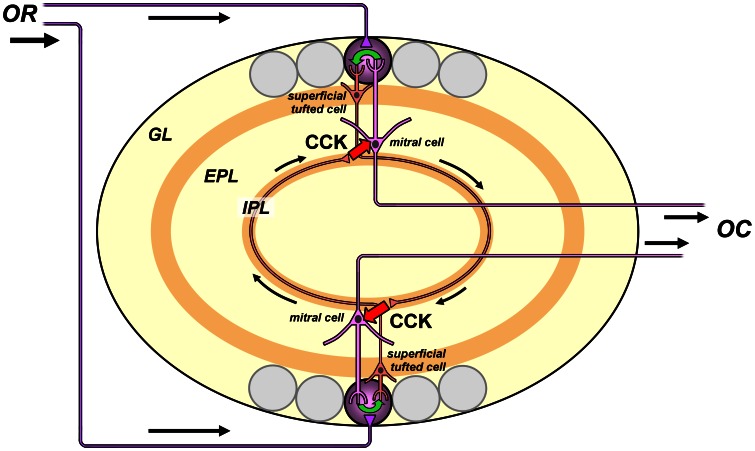
CCK-dependent positive feedback circuit for amplifying glomerular outputs. Schematic illustrating how excitation of mitral cells by CCK released from tufted cell terminals in the inner plexiform layer (IPL) might provide a critical link in a reciprocal excitatory synaptic pathway between two isofunctional glomeruli receiving inputs from the same olfactory receptor (OR). The OR inputs excite principal neurons (superficial tufted cells and mitral cells) of both glomeruli. Action potentials of superficial tufted cells of one glomerulus propagate through the IPL to the opposite side of the bulb, where they trigger release of CCK from the tufted cell terminals. The CCK migrates by extrasynaptic diffusion (RED arrows) to nearby mitral cells linked to the opposite mirror symmetric glomerulus. Mitral cells are excited by CCK, and relay this excitation to their sister superficial tufted cells by intraglomerular transmission between apical dendritic tufts (GREEN arrow). The sister tufted cells send CCK-ergic projections back to excite mitral cells linked to the opposing glomerulus. Mitral cell spikes are relayed as bulbar output to olfactory cortex (OC). This positive feedback loop may operate on slow modulatory time scales to amplify output from pairs of glomeruli connected by the intrabulbar association system. Not shown are putative glutamatergic excitatory synapses of superficial tufted cell terminals onto granule cells, which may coordinate activity of isofunctional glomeruli on faster (sniff-to-sniff) time scales. The gain of the interglomerular amplifier depends on the effectiveness of excitation by diffusional signaling (RED arrows), and might be adjustable by varying the spatial dispersion of IPL terminals through activity-dependent plasticity. Often used olfactory receptors and glomeruli responding to frequently encountered odorants are predicted have higher gain, due to efficient CCK signaling from compact IPL terminal fields. Seldom used glomeruli that only respond to rarely encountered odorants are predicted to have lower gain, due to inefficient CCK signaling from dispersed IPL terminal fields. Abbreviations: EPL, external plexiform layer; GL, glomerular layer; IPL, inner plexiform layer.

The excitation of mitral cells by CCK released from tufted cells can further boost mitral cell activity by a network amplification mechanism. Excitation between principal neurons creates positive feedback loops. We propose that such a loop operates when isofunctional glomeruli are mutually excited by reciprocal intrabulbar projections. It would include the follow steps (Fig. 10): (1) mitral cells are depolarized by CCK released from tufted terminals in the underlying IPL; (2) they transfer excitation to sister tufted cells in the same glomerulus, through intraglomerular coupling of dendritic tufts by gap junctions or glutamatergic transmission [74], [75]; (3) the sister cells relay spikes across the bulb to mirror image IPL terminals that release CCK and excite nearby isofunctional mitral cells of the second glomerulus; (4) tufted cells in this glomerulus are excited by their sister mitral cells, and they close the loop by releasing additional CCK from return projections in the original IPL terminal field. This feedback loop would amplify the activity of output neurons in pairs of glomeruli coactivated by the same olfactory receptor. The conditions for activation of this interglomerular loop depend on several unknown factors, such as how CCK release is controlled by tufted cell spiking patterns, and how CCK excitation of mitral cells might interact with more rapid inhibition received through the granule cell pathway. Such questions might be addressed by emerging optogenetic technologies that enable precise control of different synaptic pathways. The glomerular selectivity of output amplification may be degraded by crosstalk between CCK signals emitted by IPL terminals of adjacent glomeruli responding to the same odorant. However, the impact of such crosstalk could be minimized by the heterogeneity of stimulus tuning among neighboring glomeruli [76].

The feedback amplification loop that we hypothesize depends on CCK diffusion from IPL terminals to mitral cell bodies, and is hence expected to be highly sensitive to the spatial distribution of terminal projections. These projections are dynamic, exhibiting strong activity-dependent plasticity both in early development and in adult animals. Odor deprivation results in dispersion of the IPL terminal field and loss of precision in intrabulbar connections between pairs of isofunctional glomeruli, whereas odor stimulation contracts the IPL terminal field and refines the connections [23], [77]. This process might rely on the activity-dependent stabilization of synapses between tufted cells and isofunctional granule cells coactivated by the same olfactory receptors [78], [79]. If an olfactory receptor is rarely activated and its IPL terminals become more dispersed, they will excite isofunctional mitral cells less effectively due to greater distances of CCK diffusion, and the gain of the feedback amplification circuit will be lowered. If an olfactory receptor is frequently activated and its IPL terminals become more localized, they will excite isofunctional mitral cells more effectively, and the gain of amplification will be raised. Thus, the amplification of glomerular output should depend on olfactory experience and be biased in favor of receptors that bind and transduce more frequently encountered odorants. Activity-dependent stabilization of more frequently used intrabulbar projections would be further reinforced by the elevated neural activity driven by feedback amplification. We can view this as a kind of adaptive memory for tuning and optimizing bulbar processing of glomerular maps in different odor environments.
